# A multiplex PCR method to determine the sex of fetal rat tissues

**DOI:** 10.1007/s11033-025-10406-5

**Published:** 2025-03-13

**Authors:** Cristine Camp, Paige Drotos, Adrian Courville, Miranda Reed, Rachel West

**Affiliations:** 1 Anatomy, Physiology, Pharmacology Department, College of Veterinary Medicine, Auburn University, Auburn, AL USA; 2 Department of Drug Discovery and Development, Harrison College of Pharmacy, Auburn University, Auburn, AL USA; 3 Center for Neuroscience Initiative, Auburn University, Auburn, AL 36849 USA

**Keywords:** Rat, Genotyping, Genetic sex, Placenta, Fetal tissues

## Abstract

**Background:**

Fetal and placental sex influence a variety of developmental processes during prenatal life; including metabolism, growth, and the response to in utero insults. Additionally, the National Institute of Health’s requirement that sex as a biological variable be included into proposal design necessitates the development of tools to investigate sex during embryonic and fetal life. Rodent models are insightful models in the study of sexual dimorphism due to large litter sizes, short gestation period, and frequency of use as an animal model. In this methods paper, we demonstrate a multiplex PCR method to determine sex in fetal rat tail snips and placentas.

**Methods and Results:**

We designed primers for X-chromosome and Y-chromosome homologs, *DDX3X* and *DDX3Y,* and developed a single-step PCR protocol that can determine the presence of both genes in one reaction. We performed PCR on fetal tail snips and placentas to amplify *DDX3X* only in females or *DDX3X* and *DDX3Y* in males. The multiplex PCR and subsequent gel electrophoresis revealed that the presence of only *DDX3X* or both *DDX3X* and *DDX3Y* could be detected in fetal tissues. We used adult male rat testis as a positive control and confirmed that both *DDX3X* and *DDX3Y* could be detected in adult male tissues as well.

**Conclusion:**

This protocol provides an important method to determine genetic sex in tissues before the ability to visually determine sex, allowing for sex to be used as a biological variable in prenatal research using the rat model.

## Introduction

There is a growing body of work demonstrating that biological sex is a powerful influence on the development and health of humans and animals. These findings have catalyzed funding agencies to account for sex as a biological variable in grant proposals and research projects [1]. As more emphasis has been placed on better understanding sexual dimorphism, we have become increasingly aware that sex influences prenatal growth and development as well [2–4]. As there are significant ethical and scientific limitations that prevent researching the molecular and physiological events that contribute to human pregnancy, the use of rodent models is common. Humans and rodents both have a hemochorial class of placenta, defined by its invasive nature and intimate relationship with the maternal blood supply [5]. Compared to the mouse, the rat placenta has significantly deeper invasion into the placenta and trophoblast-led uterine spiral artery remodeling [6], making it an important animal model for pregnancy related research.

Determining the sex of postnatal rat pups is straightforward as sex can be identified visually by assessing the anogenital distance in pups [7]. However, this external landmark cannot be assessed in rat fetuses. Furthermore, while there are several published PCR assays to determine genetic sex in mice [8–10], there are few protocols for the rat. This creates the need for a method to identify the genetic sex of rat embryos, fetuses, and extraembryonic tissues. We have developed a single step PCR protocol to amplify the X chromosome and Y chromosome homologs, the genes *Ddx3x* and *Ddx3y.* Amplification of the X-linked *Ddx3x* serves as an internal control that can account for the female genome while amplification of the Y-linked *Ddx3y* indicates the presence of the Y chromosome. We tested our protocol in both fetal and placental tissues and were able to successfully amplify *Ddx3x* in male and female fetal tail snips and placentas and *Ddx3y* in only male fetal tail snips and placentas.

## Materials

**Table:** 

Reagent	Concentration	Company/catalog number	Comments
DNA Isolation			
Lysis Buffer			
Nonidet P-40 Substitute	0.3%	Amresco, M158-100 mL	
Potassium Chloride (KCl)	50 mM	VWR, BDH9258-500G	
Tris	10 mM	VWR, 97,061–794	pH to 8.3
Tween20	0.3%	Fisher Scientific, BPBP337500	
Proteinase K	1 mg/mL	Boston BioProducts, P-1460	
PCR Reaction			
*Ddx3x* Fwd Primer	10 µM		
*Ddx3x* Rev Primer	10 µM		
*Ddx3y* Fwd Primer	10 µM		
*Ddx3y* Rev Primer	10 µM		
Molecular biology grade water		VWR, VWRL0201-0500	
2X Phusion U Green supermix		Fisher Scientific, F-564	
1X TE Buffer		Invitrogen, AM9849	pH 8.0
Gel Electrophoresis			
FluorStain		SMOBio, DS1000	
GeneRuler 100 bp Plus DNA Ladder		Thermo Scientific, SM0323	
RA Agarose		Amresco RA, N605-500G	
TAE Buffer		VWR, K915-1.6 L	50x
Tritrack 6X Loading Dye		Thermo Scientific, R1161	
Gel Imaging			
ChemiDoc		Bio-Rad	

## Methods

### Primer design

We obtained genomic sequences for *Ddx3x* (NC_086039.1) and *Ddx3y* (NC_086040.1) on NCBI Gene. FASTA sequences were used to design primer sequences using the Primer3web software (primer3.ut.ee). Amplicon size ranges were chosen between 150 and 250 base pairs for *Ddx3y* and 250–350 base pairs for *Ddx3x.* The sequences for *Ddx3x* and *Ddx3y* are as follows; *Ddx3x* Forward primer: 5′ – GCATGCCCGCCTACAATTTA – 3′, *Ddx3x* Reverse primer: 5′ – CCACGGCTGCTACCCTTATA – 3′, *Ddx3y* Forward primer: 5′ – AGCAGTTTTGGATCTCGGGA – 3′, *Ddx3y* Reverse primer: 5′ – TCTGTCCAGCCCCAAGATAC – 3′. Sequences can also be seen in Table 1.

**Table 1:**
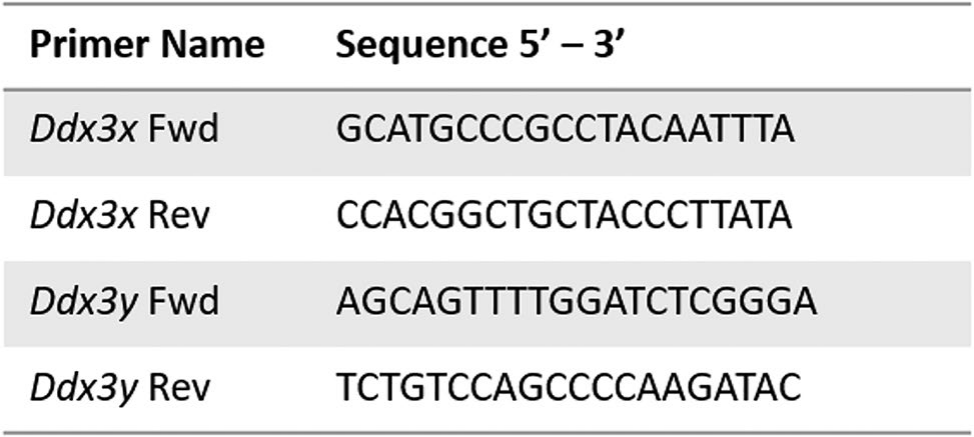
*Ddx3x* and *Ddx3y* primers

Primer sequences used to amplify *Ddx3x* and *Ddx3y*

### DNA isolation

1. Pre-heat two heating blocks, one to 55 °C and one to 98 °C.
2. Mix 96 µL of Lysis Buffer with 4 µL Proteinase K in a microcentrifuge tube.
3. Add tail snip or placental tissue to microcentrifuge tube containing Lysis Buffer + Proteinase K.
4. Vortex briefly and place tube into 55 °C heat block for 1 h.
5. After 1 h, move microcentrifuge tube to 98 °C heat block and incubate for 15 min to inactivate Proteinase K.
6. After 15 min, spin microcentrifuge tube at 2,000 × g for 3 min at room temperature to separate undigested debris.
7. Move supernatant to a fresh microcentrifuge tube.
8. At this point, sample can be frozen at −20 °C for long term storage or 4 °C for short term storage.

### Multiplex PCR

1. Dilute DNA to 20–50 ng/uL using molecular biology grade water.
2. Make primer mix by adding 10 µM *Ddx3x* forward and reverse primers and 10 µM *Ddx3y* forward and reverse primers at a 1:1 ratio.
  - Primers should have previously been reconstituted to 100 µM stock in 1X TE Buffer and then diluted to a 10 µM working solution using molecular biology grade water.
3. For each PCR reaction add:
  - 10 µL of 2X Phusion U Green Supermix
  - 10 µL molecular biology grade water
  - 1 µL of primer mix
  - 1 µL of diluted DNA sample
4. Briefly vortex and centrifuge PCR tubes.
5. Place tubes into thermal cycler and use the following PCR protocol:

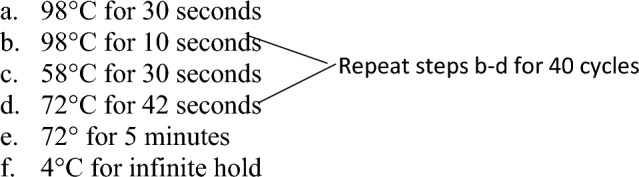

6. Dilute PCR product 1:5 in molecular biology grade water before moving to gel electrophoresis.

### Agarose gel electrophoresis

1. Make a 3% gel using 3 g of RA agarose and 100 mL of 1X TAE buffer.
2. Add 10 µL DNA fluorostain dye.
3. Load 5 µL 100 bp ladder.
4. Mix 10 µL diluted PCR product with 2 µL loading dye.
5. Load 10 µL of the mixture into each well of gel.
6. Run gel at 4 °C at 50 V until sufficient separation is achieved.
7. Immediately image gel.

## Results

### Presence of *Ddx3x* and *Ddx3y* in male and female fetal tail snips and placentas

**Fig. 1:**
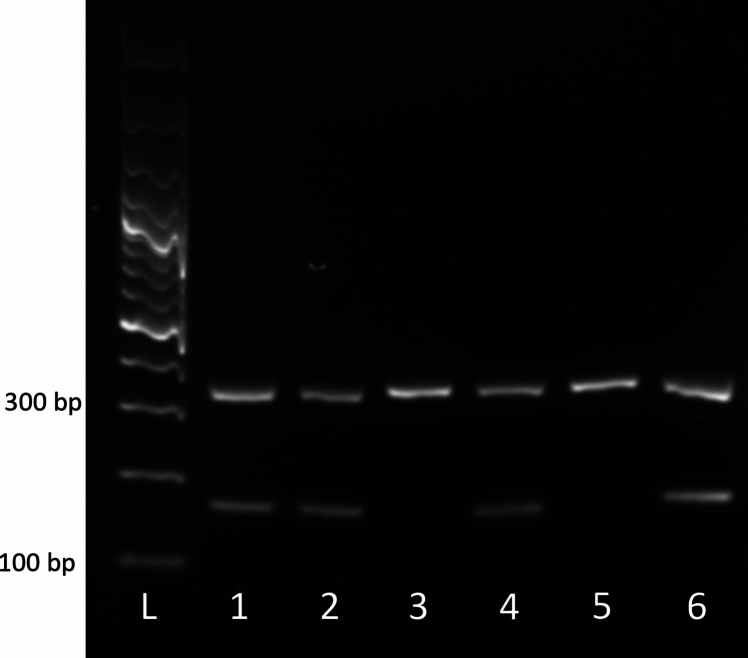
PCR products of rat placental tissues. *L* – 100 bp ladder, *1* – Adult testis, *2* – Adult testis, *3* – female placenta, *4* – male placenta, *5* – Female fetal tail snip, *6* – Male fetal tail snip. Amplicon at 320 bp is *Ddx3x*. Amplicon at 180 bp is *Ddx3y*

The presence of a Y chromosome was examined using primers for the Y-linked gene *Ddx3y*. As an internal control, primers for *Ddx3x* were used with each sample. After performing PCR, we ran the amplified DNA on an agarose gel using electrophoresis. The male fetal tail snip and placentas had both X-chromosome specific and Y-chromosome specific amplicons as evidenced by the presence of two bands, one for *Ddx3x* (amplicon size 320 bp) and one for *Ddx3y* (amplicon size 185 bp) **(**Fig. 1**)**. The female fetal tail snips and placentas had the *Ddx3x* X-chromosome specific amplicon, indicating that the Y chromosome is not present in these samples (Fig. 1**)**.

### Troubleshooting

#### Failure to amplify both *Ddx3x* and *Ddx3y* bands in male samples

A problem we encountered early in the development of this protocol is the presence of only the *Ddx3y* amplicon of male samples in the multiplex PCR protocol. However, when PCR was performed for each gene independently, amplicons for both *Ddx3x* and *Ddx3y* would appear in their individual lanes. To overcome this, we tested different annealing temperatures and found that 58 °C was the ideal annealing temperature to detect the presence of both amplicons using a multiplex PCR reaction.

Additionally, we tried several different master mix recipes, concentrations of primers, and concentrations of template for the single-step PCR reaction. Multiplex PCR requires a delicate balance of magnesium chloride, deoxynucleotide triphosphates (dNTPs), buffers, primers, template DNA, and DNA polymerases [11]. We were most successful using the commercially available high-fidelity Phusion U Multiplex PCR master mix provided by Thermo Scientific.

## Discussion

In this methods paper, we provide a simple single-step multiplex PCR protocol to determine genetic sex in fetal rat tissues. Historically, determination of genetic sex by PCR has been performed by amplifying the Y-chromosome specific gene, *Sry.* However, solely amplifying for the presence of *Sry* leaves the user without a proper internal control. By amplifying both *Ddx3x* and *Ddx3y*, we have created a protocol that tests for the presence of the Y-chromosome while using the X-chromosome as an internal control, ensuring the user that their method is sound by removing the possibility of a false negative. Additionally, by including placental and adult male testis tissue, we have demonstrated that both fetal and adult tissues that are not traditionally used for genotyping can be successfully used to amplify *Ddx3x* and *Ddx3y.* This is useful for researchers who have tissues frozen but are unsure of the sex and would like to carry out experiments considering sex as a biological variable.

We acknowledge that there is at least one other publication that provides a protocol to determine genetic sex in rat tissues [12]. However, in our hands, we were unable to amplify both genes in one reaction. In this paper, we selected two different X- and Y- chromosome homologs and designed new primers. Our proposed method provides an alternative approach to determine genetic sex in rat tissues using a multiplex PCR method.

## Conclusion

To summarize, we have developed a simple, cost-effective method to determine the genetic sex of rat fetal and placental tissues using multiplex PCR.

## Data Availability

No datasets were generated or analysed during the current study.
